# The success of transdisciplinary research for sustainable land use: individual perceptions and assessments

**DOI:** 10.1007/s11625-018-0556-3

**Published:** 2018-03-28

**Authors:** Jana Zscheischler, Sebastian Rogga, Andrej Lange

**Affiliations:** 1 Leibniz Centre for Agricultural Landscape Research, Research Area “Land Use and Governance”, Eberswalder Str. 84, 15374 Müncheberg, Germany; 2 Centre for Technology and Society (ZTG), Technische Universität Berlin, Hardenberg Str. 16-18, 10623 Berlin, Germany

**Keywords:** Cooperation, Quality measurement, Evaluation, Research evaluation

## Abstract

Evaluation is a major issue in discussions of transdisciplinary research (TDR). Empirical studies often consider expert perspectives; however, knowledge of the experiences, attitudes, and motivations of a broader science-practice community applying transdisciplinarity remains rare. The present study aims to gather insights into the perceptions and assessments of success of TDR projects from scientists and practitioners with experience with TDR processes. Based on a mixed-method approach combining qualitative expert interviews with a quantitative survey reaching 178 respondents from practice and science, the results show a high commitment to the targets of TDR projects and a basic shared ‘success profile’. Nevertheless, there is currently a strong ‘practice tendency’, while TDR-specific benefits of the scientific knowledge gain remain neglected. The general success assessment of TDR projects can be described as rather moderate, indicating several deficits in the application and management of TDR.

**Electronic supplementary material:**

The online version of this article (10.1007/s11625-018-0556-3) contains supplementary material, which is available to authorized users.

## Introduction

With the increasing introduction of transdisciplinary research (TDR) in sustainability sciences (Komiyama and Takeuchi 2006), the issue of evaluation has come to the fore. Today, a large part of the literature is dedicated to the search for adequate evaluation approaches (e.g., Carew and Wickson 2010; Walter et al. 2007; Wickson et al. 2006; Roux et al. 2010; Jahn et al. 2012; Klein 2008). However, Klein (2008) claimed that the evaluation of TDR is ‘one of the least-understood aspects’, and this still can be argued to be true. In the search for consistent evaluation criteria for TDR, special difficulties are discussed that are caused by the context specificity of TDR, unforeseeable changes in the course of a project, the high degree of uncertainties, and the lack of comparability between different projects (Wickson et al. 2006). In addition, measurement of the societal (‘real-world’) impact of TDR poses a major and thus far unsolved challenge that is made difficult because of non-linear interdependencies, multiple interacting drivers, and long-effective periods (Roux et al. 2010).

Currently, research evaluation is dominated by reductionistic procedures that are based on few quantitative indicators and, thus, are unsuited to adequately display TDR achievements (see also Walter et al. 2007; Kaufmann and Kasztler 2009; Wolf et al. 2013). As a result, researchers who join and commit themselves to collaborative research projects such as transdisciplinary ones must fear that they will suffer disadvantages involving their reputation and funding opportunities (Wolf et al. 2013).

Moreover, TDR is ‘not uncontested outside the transdisciplinary research community’ (Lang et al. 2012). Thus, on one hand, there is a need to prove and secure the promised societal benefits (de Jong et al. 2016; Walter et al. 2007; Spaapen et al. 2007; Stokols et al. 2003; Blackstock et al. 2007), whereas, on the other hand, the scientific benefits (Hegger and Dieperink 2015) and the impact of TDR publications (Stipelman et al. 2014) must be clarified.

The scientific literature on TDR evaluation primarily identifies two foci. One group of scholars focuses on the question of how to measure the impact of TDR (e.g., Walter et al. 2007; Spaapen et al. 2007; Blackstock et al. 2007). A second (larger) group seeks to develop and discuss adequate sets of quality criteria, emphasizing the importance of quality assurance through self-reflection and learning. These attempts use various arrangements and have different emphases: Lang et al. (2012) organize different principles of TDR along a 3-phase model, whereas Belcher et al. 2016 sort several criteria along the following four principles: relevance, credibility, legitimacy, and effectiveness. Jahn and Keil 2015 emphasize the different perspectives and needs of different actor groups and have developed actor-specific guidelines to support program managers, researchers, and policymakers. Wickson and Carew (2014) provide orientation through a set of paired criteria that are broad and open to interpretation, which can be complemented by individual indicators specific to every case. Long sets of guiding questions for formative self-evaluation are found in the approaches of Bergmann et al. (2005) and Defila and DiGiulio (1999).

All these efforts have contributed to further consolidation of the often unclear and elusive concept of TDR, as they provide guidance and benchmarks for developing and conducting TDR projects (Belcher et al. 2016; Jahn and Keil 2015; Wickson and Carew 2014; Hessels et al. 2014). Still, no generally accepted standards have been established. Some scholars argue that the absence of clear guidelines results in ‘muddling through’ and hinders the development and proliferation of TDR (Klein 2008; Jahn and Keil 2015; Belcher et al. 2016). Jahn and Keil (2015) assume that a ‘plurality of TDR’ and the lack of a generally applicable definition may explain the absence of commonly agreed upon standards. Currently, there seems to be a basic agreement that TDR projects vary in many aspects, and thus, a ‘broad adaptable set of criteria’ is needed (Wolf et al. 2013; Wickson and Carew 2014; Belcher et al. 2016).

In addition, contributions to criteria sets are prevailingly related to an ideal-typical concept of TDR (Bergmann et al. 2005; Lang et al. 2012; Jahn 2014) and are mainly driven by TDR-advocating scientists. As known from the previous contributions (see Fuest and Lange 2015; Scholz and Steiner 2015; Blättel-Mink and; Kastenholz 2005; Zscheischler et al. 2017), a gap between the ‘idealized’ concept of transdisciplinarity and ‘real-world’ practice is evident. Moreover, the criteria sets which we found were developed by and for TDR experts. In contrast, knowledge of the experiences, attitudes, and motivations of a broader science-practice community applying transdisciplinarity remains rare (Schmid et al. 2016). Little is known about how satisfied researchers and stakeholders are with TDR projects and how they assess the general project success related to which criteria. Stakeholders’ perspectives remain widely unconsidered. Because the TDR approach is still in the process of diffusion to a wider science-practice community, the assessment of TDR by its applicants may be of special importance.

Against this backdrop, a quantitative measurement of success by all participants represents an opportunity to depict the subjectively perceived quality of TDR projects. The assessment by all project participants poses special requirements for applied criteria such as understandability and measurability. Because evaluation and related criteria depend also on the definition of TDR, which may vary widely (e.g., Klein 2008; Jahn and Keil 2015), the assessment may also provide insights into the understanding of TDR.

In the project management literature, the concept of ‘success’ is discussed as a ‘multidimensional construct interrelating technical, economic, behavioral, business, and strategic dimensions’ (McLeod et al. 2012). Project success is considered a social phenomenon constructed subjectively and intersubjectively by individuals and groups of individuals and reflecting goal achievements, attitudes, motivation structures, and satisfaction (Ika 2009; Alderman and Ivory 2011; McLeod et al. 2012). Hence, measuring project success means also accounting for actors’ different views (Stuckenbruck 1986 cit. after; Atkinson 1999; McLeod et al. 2012). Thus, the concept of success might help reveal differences not only in individual perceptions but also in interests, beliefs, motivations, and conflicting targets. Because balancing and integrating multiple competing stakeholder goals and inherent conflicts appear to be specific challenges of TDR practice (e.g., Zscheischler et al. 2014), focusing the concept of ‘success’ could be a promising approach for revealing contradictions and inherent conflicts within TDR projects.

The aim of this paper is to give insights into the success perceptions and assessments of TDR from scientists and practitioners experienced in TDR projects in the field of land-use science. For this purpose, the results of qualitative interviews and a survey with scientists and practitioners are introduced and discussed. The following questions are addressed: What defines a successful TDR project? What is the overall perceived project success, and how is it associated with different criteria of success?

There are different notions of TDR, and a commonly shared definition is still lacking (see also Pohl and Hirsch Hadorn 2006; Brandt et al. 2013; Jahn and Keil 2015) not only because of regional differences (Klein 2008; Zscheischler and Rogga 2015). Even though pluralities abound, core characteristics that describe a shared set of features can be found. In accordance with numerous authors (e.g., Scholz 2011, Jahn et al. 2012, Pohl and Hirsch Hadorn 2008, Lang et al. 2012), we define TDR as a reflexive collaborative research approach that integrates knowledge and perspectives from different disciplines and stakeholders; it facilitates mutual learning processes and aims to contribute to solutions for complex real-world problems.

## Methods

For this study, we collected data on perceptions and assessments about success as well as the corresponding success criteria for TDR using a two-step approach. First, we conducted explorative qualitative interviews with coordinating scientists of ten TDR projects, where our aim was to gain information on the perceived benefits of the project and the underlying evaluation criteria. Building on these insights, in the second step, we developed and conducted a Web survey to obtain quantitative data on perceptions and assessments from researchers and practitioners who have participated in a TDR project.

### Interviews

The interviews focused on qualitative aspects in the assessment of the success of TDR projects. The aim was to gain information on the variety and range of success perceptions and the corresponding explanatory criteria. For this purpose, we asked coordinators of 10 finished TDR projects to assess the success of their last TDR project and to judge their assessment by relevant criteria. The interviews were conducted between September 2015 and December 2015, and they lasted up to 1 h and were recorded and fully transcribed. As we were members of a scientific coordination project that was set up to accompany the TDR projects over the entire funding period, we had particularly good access to all ten projects. We got the opportunity to participate in numerous informal talks, which complemented insights from documents and many meetings in each project.

Using the software MaxQDA, we coded and analyzed all interviews by following the steps of qualitative content analysis in Mayring (2008). The criteria that were identified as important for a successful TDR project by the interviewees served as the basis for the answer categories of the closed-ended questions posed in the online questionnaire.

### Online survey

An online survey was used to gather information on the perceived success and corresponding criteria from a broader range of scientists and practitioners. Our target group was participants recently involved in TDR projects in the context of land-use issues. Invitations to participate in the survey were sent to 438 researchers and practitioners known from 21 recently finished TDR projects. All invitations were sent via e-mail and contained an access key, and after 2 weeks, an e-mail reminder was sent. We posted the survey from October 2016 until November 2016.

Overall, 178 participants completed the questionnaire. The overall response rate was thus about 41%. The online questionnaire started with a filtering question asking respondents whether they had participated in a TDR project. Respondents answering ‘no’ to this question were excluded from the analysis. Finally, answer sets from 164 respondents (119 scientists and 45 practitioners) remained for analysis.

Most of the survey questions were closed and designed to be answered on a five-point scale.

#### Surveying the perceived success and corresponding criteria

Respondents were asked to answer all questions with reference to their most recent TDR project. In the first step, we asked them to qualify what constitutes a successful TDR project on the basis of 14 items. These items were synthesized from the codings of the interviews with coordinators (see Table 3) and complemented by further TDR-specific criteria obtained by reviewing the relevant literature.

Table 1 shows the items used for the survey and their connections to Table 3. The selection was determined by several requirements: (i) a manageable number of answer categories was needed to ensure an appropriately high response rate; (ii) the answer categories had to be applicable on a broad range of TDR projects; and (iii) the answer categories also had to be understandable and answerable by scientists and practitioners. Some items were adopted almost identically and others were summarized or added. Added items reflect qualitative aspects of collaboration (7, 12), proxies of scientific practice (8, 14), and the question of relevance to sustainability (11).

**Table 1:** Items used to survey the importance of individual success criteria (numbers in brackets reference connections to categories from qualitative interviews, see Table 3)

Question: What characterizes a successful transdisciplinary research project?	
1	The project develops implementable solutions for practice (24, 13)
2	A follow-up project can be acquired (11)
3	Mutual learning processes take place between science and practice (3, 4, 5)
4	The project produces high scientific publication output (7)
5	Popularity of the project in the corresponding expert community can be achieved (9, 14)
6	Results from sub-projects merge into an overall synthesis
7	Representatives from all important stakeholder groups are involved
8	During the project cycle, doctoral theses can be conducted
9	Project results are generalizable and transferable to other contexts (17)
10	Scientific knowledge can be gained (15)
11	The project significantly contributes to the more sustainable use of natural resources
12	Science-practice cooperation takes place on an equal basis
13	Results are implemented in practice (13)
14	New scientific methods and theories are developed

A five-point scale was provided to rate each item (1 = ‘not important’, 5 = ‘very important’). The aim was to find a preferred ‘success’ profile.

In the second step, we asked respondents to assess the overall success of their recently finished TDR project on a five-point scale (1 = ‘not successful at all’, 5 = ‘very successful’) as a single survey item. Then, in the third step, we asked them to again rate the 14 items regarding the achievements of their recent TDR project (1 = ‘strongly disagree’, 5 = ‘strongly agree’). Within all five-point scales, we additionally provided a “don’t know” option.

To reveal which project characteristics affect the perception of overall project success, we used socio-demographic attributes and indicators for the quality and quantity of participants’ professional experience (see Table 2). We then analyzed the survey data (*N* = 164) in several steps. First, we applied descriptive statistics to order the 14 items according to their importance rating for an ideal TDR project and to calculate the perceived overall success of the project and the item ratings regarding the respondents’ recent TDR projects. Using non-parametric procedures (Mann Whitney *U* test and Kruskall and Wallis test), we tested for differences between independent samples for the independent variables as listed in Table 2. In the second step, we compared the rating of the ‘ideal-type’ project with the rating for the recent project using the Wilcoxon test. The Wilcoxon test is a non-parametric test which shows whether central tendency of two dependent samples differ. The aim was to reveal strengths and weaknesses in TDR practice. To ascertain relations between these criteria and overall success perceptions, we generated a correlation matrix. The result showed a diverse image of correlations split between the ratings for practice and the ratings for science. To identify interactions between the ratings of items and gain a better interpretation, in the last step, we undertook a categorical principal components analysis.

**Table 2:** Independent variables used to analyze influences on the perception of success in transdisciplinary research projects

No.	Categories and independent variables
1	Age (< 29, 30–39, 40–49, > 50)
2	Gender (female/male)
3	Profession (practice/science)
4	Disciplinary background (nature sciences, engineering sciences, social sciences, economic sciences, humanities and cultural sciences, and legal sciences)
5	Professional experience [number of transdisciplinary research projects in which respondents participated (1, 2, 3–5, 6, or more)]

#### Sample characteristics and access

The qualitative and quantitative data for this study were collected by members of a scientific coordination project (SCP) that accompanied many of the selected TDR projects over a period of 6 years, starting in 2010. This setup provided valuable insights into each research project.

All projects took place in different areas of Germany and were part of the same funding program, which aimed to develop sustainability solutions for land-use related challenges. Project objectives included the development of innovative value creation networks for sustainable regional development, new instruments and concepts of resource efficiency for settlement development, decentralized systems of renewable energies, and new technologies supporting sustainable land-use systems. Application of the TDR approach was a prerequisite for funding. The call for proposals demanded the integration of various scientific disciplines and the involvement of actors such as ‘decision makers’ and ‘key actors’. In the field of land use, practitioners were mainly professional experts from local, municipal, and regional administrations (spatial planning, water, forestry, etc.), small- and medium-sized enterprises (farmers, municipal energy suppliers, etc.), professional associations, and consultancies. Non-professional local and regional stakeholders (i.e., residents, civil society organizations, tourists, etc.) had also been incorporated into research activities, but to a far lesser extent. Within the sample, we differentiate between ‘scientists’ and ‘practitioners’, referring to each individual’s professional affiliation at the point of the investigation.

**Fig. 1:**
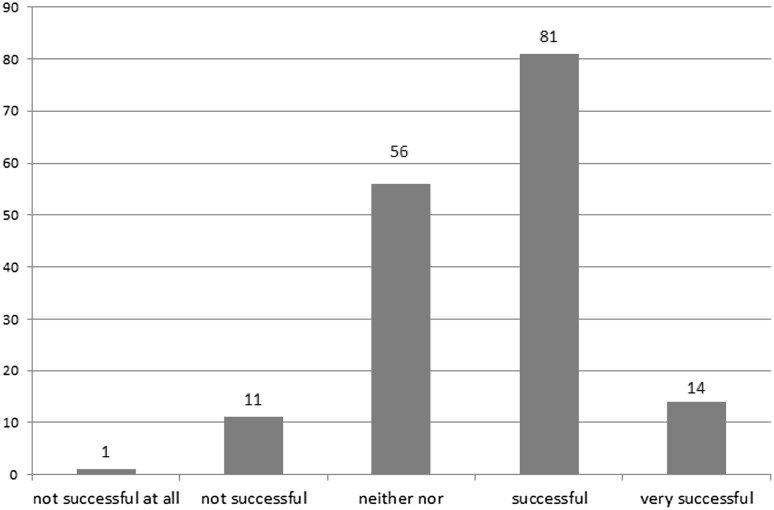
Frequency distribution of ‘overall project success’ ratings

At the scientists’ site, engineering and natural science disciplines were prevalent among the projects, which is supported both by the surveyed data and by the observations from the SCP. Approximately one-quarter of respondents can be categorized to either the social or the economic sciences (see Suppl. Data, Fig. 1), whereas the majority have a background in technical and natural scientific disciplines.

**Fig. 2:**
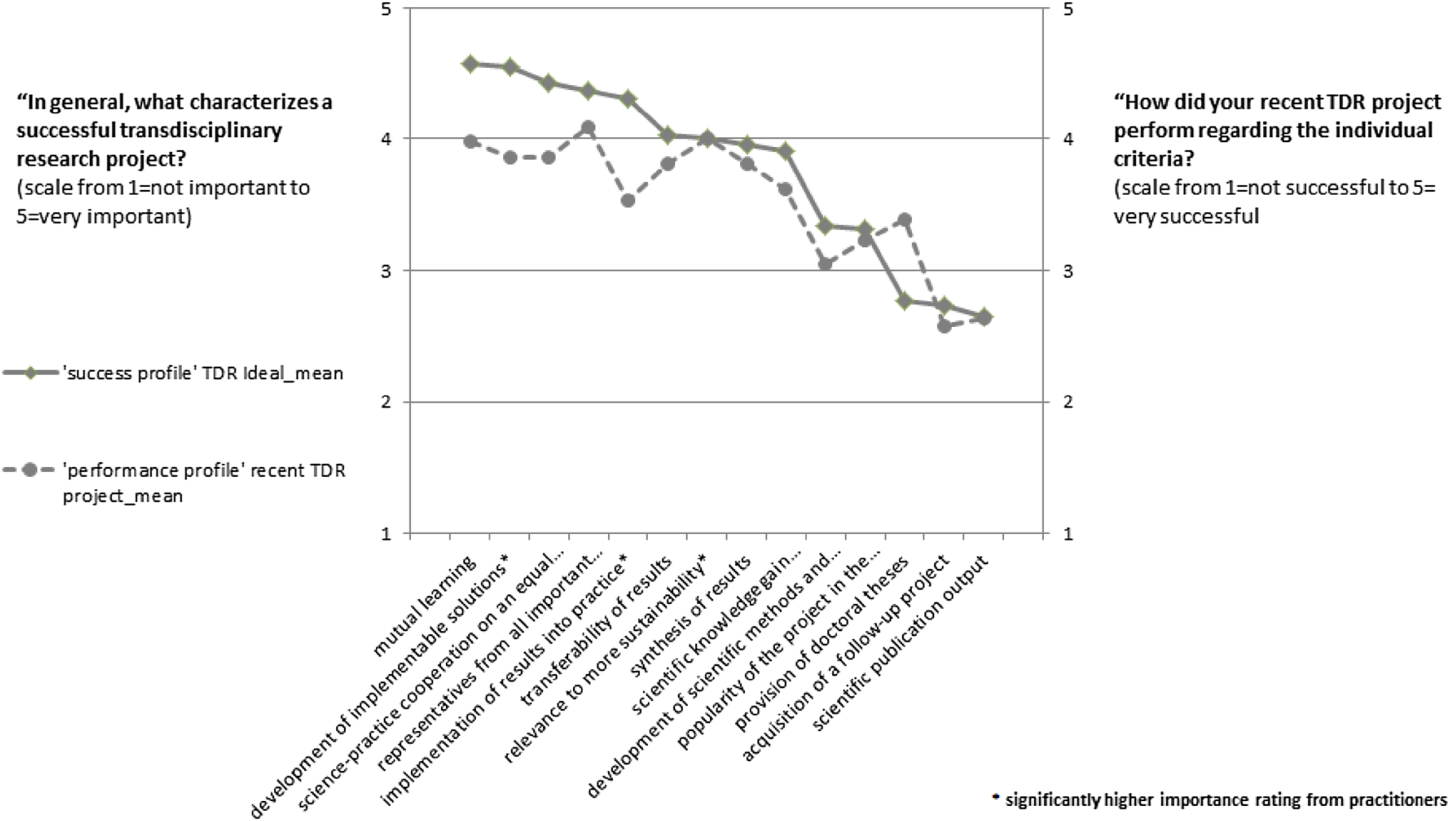
‘Success profile’ of an ideal-type TDR project resulting from the average importance ratings (continuous line)and the ‘performance profile’ of the recent TDR project resulting from the average success ratings (dashed line)

The age structure of the sample shows a high share of experienced project partners. Forty percent of the respondents were more than 50 years of age (see Suppl. Data, Fig. 3). More than half of the respondents had participated in more than three TDR projects (see Suppl. Data, Fig. 2).

## Results

In this section, we first present the results from the interviews with coordinating scientists from 10 TDR projects. Subsequently, we report the results from the questionnaire.

### Success assessment and dimensions of success as expressed in the interviews

In the first step, we asked the interviewees to estimate the specific benefits of their TDR project in comparison with other research approaches. In the interviews, we observed that respondents had difficulty answering this question: one interviewee completely negated specific benefits with regard to a lack of transdisciplinarity in his project, whereas others were confused about this issue, as exemplified by the fact that they either took long pauses before answering or repeated the question [Q1, Q2].

In the second step, all interviewees were asked to give an assessment of the overall success of their TDR project. Here, respondents had similar difficulty answering. In particular, the impacts of the projects were hardly assessable owing to many unforeseeable and external influential factors. Even in cases where desirable outcomes could have been observed, a direct mono-causal connection with the project could not have been proven [Q3, Q4].

Perceptions of the overall success of a TDR project were predominantly associated with practical relevance. Thus, the overall project success was mainly judged by the effects in practice. In general, the coordinators show a high degree of satisfaction with regard to project achievements. However, some note that the perception of project success might vary within one project or may even change over the duration of a project [Q5–Q7]. In particular, the retrospective assessments would be more positive than the perceptions during the process.

All the interviewees reported a variety of criteria reflecting the benefits of TDR when judging the success of their project. Table 3 provides an overview of the referenced criteria, which initially indicates a strong orientation toward practice. By contrast, a few items reflect the conventional scientific success criteria. Notably, the mentoring and support of scientific junior staff, which is a core criterion of scientific excellence, was not mentioned at all.

**Table 3:** Dimensions of success as expressed in selected interviews

Question: What defines a successful transdisciplinary research project?	
1	Dealing with conflicting issues/resolving conflicts and resistance
2	To win the attention of practitioners/to stimulate enthusiasm
3	To initiate a successful course of discussion
4	Learning processes/new insights
5	To show feasibilities and new possibilities to practice
6	To establish acceptance
7	Publication output
8	Number of events
9	Popularity of the project in the corresponding expert community
10	Collaboration
11	Follow-up projects
12	Continuity
13	Implementation of results into practice
14	To increase the popularity of the issue
15	Scientific knowledge gain/‘Erkenntnisgewinn’
16	To generate products and tools
17	Transferability of results
18	Encouragement from practitioners
19	An agreed and accepted result
20	Project awards
21	Quality of research reports
22	Internationalization
23	To be on schedule
24	‘If you find solutions for the problems of practice’

### Success assessment and dimensions of success as revealed by the online survey

#### What defines a successful TDR project?

To answer the question of what defines a successful TDR project, we asked respondents to rate the importance of 14 success criteria for TDR. These criteria were gathered and developed on the basis of interviews with coordinators, as described above (see also Table 3).

Table 4 presents the assessment of success criteria in descending order of importance (from high to low mean value). As shown, typical criteria reflecting the cooperation with and relevance for practice, such as ‘mutual learning’, ‘development of implementable solutions for practice’ and ‘science-practice cooperation on an equal basis’ were rated as the most important criteria for success, and the relatively low variance values indicate basic agreement on these criteria.

**Table 4:** Importance of individual criteria for the success of a transdisciplinary research project as rated in the online survey (TDR success profile)

No.	Question/item ‘In general, what characterizes a successful transdisciplinary research project?’ (scale from 1 = not important to 5 = very important)	*N*	Min	Max	Mean	Std. dev.	Variance
1	Mutual learning	163	1	5	4.5767	0.61760	0.381
2	Development of implementable solutions for practice*	161	1	5	4.5404	0.72452	0.525
3	Science-practice cooperation on an equal basis	162	1	5	4.4198	0.79383	0.630
4	Representatives from all important stakeholder groups are involved	162	1	5	4.3580	0.80847	0.654
5	Implementation of results into practice*	159	1	5	4.2830	0.85041	0.723
6	Transferability of results	160	1	5	4.0438	0.93396	0.872
7	Relevance to more sustainability*	153	1	5	4.0131	1.03864	1.079
8	Synthesis of results	161	1	5	3.9689	0.99638	0.993
9	Scientific knowledge gain (‘Erkenntnisgewinn’)	160	1	5	3.9250	0.89408	0.799
10	Development of scientific methods and theories	161	1	5	3.3354	1.04250	1.087
11	Popularity of the project in the corresponding expert community	162	1	5	3.3333	1.01541	1.031
12	Provision of doctoral theses	160	1	5	2.8063	1.10742	1.226
13	Acquisition of a follow-up project	156	1	5	2.7115	1.185991	1.407
14	Scientific publication output	160	1	5	2.6750	1.06724	1.139

*Significantly higher importance rating from practitioners

In contrast, typical indicators for scientific excellence, such as ‘scientific publication output’, ‘acquisition of a follow-up project’, and the ‘provision of doctoral theses’ were rated as rather less to not important.

Testing for differences between independent sample groups revealed significant differences for a few items. The Mann–Whitney *U* test indicates differences for the central tendency of ratings between practice and science regarding the items ‘development of implementable solutions for practice’, ‘implementation of results into practice’, and ‘relevance to sustainability’ (marked with *). For these items, practitioners tended to indicate higher importance than scientists. In addition, age influenced the rating for some items (Kruskal and Wallis Test). While the youngest researcher group and the ‘50 plus’ group reported higher ratings for the importance of possibilities to conduct a doctoral thesis, the ‘middle agers’ (30–49) considered this criterion to be less important. In contrast, respondents’ age significantly correlated with the importance rating of the item ‘sustainability’ with the ‘50 plus’ group reporting significantly higher rating than their younger colleagues.

Other independent variables (gender, age, disciplinary background, and professional experience) did not show any significant differences between independent sample groups.

Figure 2 shows the average importance ratings in the form of the continuous trendline, which we call the ‘success profile’.

#### What is the overall perceived success?

In the second step, we asked respondents to assess the overall project success of their recent TDR project. This overall project success was assessed with a mean value of 3.6 (see Table 5). Frequency distribution revealed a high share of respondents (58%) who rated their recent TDR project as either ‘successful’ (*n* = 81) or ‘very successful’, whereas another high share of respondents (42%) rated it as either ‘neither nor’ (*n* = 56) or ‘not successful’ (*n* = 12) (Fig. 1).

**Table 5:** Assessment of overall success regarding the recent transdisciplinary research project

	*N*	Min	Max	Mean	Median/modus	Std. dev.	Variance
Perceived overall success	155	1	5	3.5806	4	0.77183	0.596

The Mann–Whitney *U* test revealed significant differences in perceptions between respondents from practice and respondents from science. Specifically, respondents from practice (*p* = 0.037) tended to assess the overall project success as significantly lower (median/modus = 3) than scientists (median/modus = 4).

Other independent variables (gender, age, disciplinary background, and professional experience) did not show any significant differences between the independent sample groups.

#### How did respondents assess the performance of their projects compared to the ideal TDR project?

In the next step, we asked respondents to rate the how 14 criteria performed in their most recent TDR project, i.e., if the criteria were deemed successfully fulfilled on a 5-point scale (1 = not successful; 5 = very successful).

All but two criteria were assessed as above average. The best-performing criteria were ‘representatives from all important stakeholder groups are involved’ (4.09), ‘mutual learning’ (3.98) and ‘relevance to more sustainability’ (3.99). The two criteria on the less successful part of the scale were ‘acquisition of a follow-up project’ (2.57) and ‘scientific publication output’ (2.63).

Figure 2 shows the comparison between the perceived importance of success criteria and the actual performance of these criteria in their respective TDR projects. The graph shows two general trends. First, the criteria scored higher on the 5-point importance scale than on the corresponding 5-point scale for success—with the notable exception of the criterion ‘provision of doctoral theses’. Second, there is a correlation between the two variables: on average, items that score higher on the importance scale are also rated as performing more successfully in the respondents’ most recent TDR project.

Thus, we could find significant correlations for almost all the paired criteria except ‘mutual learning’, ‘development of implementable solutions’, and ‘implementation of results into practice’ (see Table 6).

**Table 6:** Statistically significant associations between the importance ratings (“success profile”) and the performance ratings

No.	Criteria/items	Correlation *r* (*p* < 0.01)
1	Mutual learning	–
2	Development of implementable solutions	–
3	Science-practice cooperation on an equal basis	0.443
4	Representatives from all important stakeholder groups are involved	0.461
5	Implementation of results into practice	–
6	Transferability of results	0.420
7	Relevance to more sustainability	0.254
8	Synthesis of results	0.273
9	Scientific knowledge gain (“Erkenntnisgewinn”)	0.247
10	Development of scientific methods and theories	0.414
11	Popularity of the project in the corresponding expert community	0.303
12	Provision of doctoral theses	0.373
13	Acquisition of a follow-up project	0.317
14	Scientific publication output	0.253

Associations have been analyzed using Spearman’s rank-order correlation. Non-significant associations (indicated by ‘–’) are, for reasons of clarity, not depicted

#### Which criteria affect the assessment of overall success?

To determine which criteria affect the assessment of overall success, we conducted a correlation analysis following Spearman. The results (presented in Table 7) show significant correlations between many item ratings and the assessed overall project success. Yet, the correlation pattern differs between practitioners and scientists. Noticeably, regarding practitioners, both criteria that were previously rated as especially important, ‘implementation of results into practice’ and ‘development of implementable solutions for practice’, did not show an association with the overall success assessment. Instead, two other criteria, ‘popularity of the project’ and ‘acquisition of a follow-up project’, which were previously rated as relatively unimportant, showed high correlations.

**Table 7:** Statistically significant associations between the assessment of the overall success and the success criteria

No.	Success criteria/items	In total		Practice		Science	
		*N*	*p* < 0.05	*N*	*p* < 0.05	*N*	*p* < 0.05
1	Mutual learning	159	0.299**	33	0.492**	119	0.212*
2	Development of scientific methods and theories	153	–	28	–	118	–
3	Scientific publication output	149	–	27	–	116	–
4	Popularity of the project in the corresponding expert community	148	0.263**	27	0.585**	114	–
5	Implementation of results into practice	156	0.443**	32	–	117	0.493**
6	Synthesis of results	150	0.236**	33	–	111	–
7	Acquisition of a follow-up project	130	0.285**	25	0.518**	99	0.213*
8	Representatives from all important stakeholder groups are involved	158	–	33	–	118	0.218*
9	Provision of doctoral theses	152	0.174*	29	–	117	–
10	Transferability of results	158	0.317**	32	–	119	0.323**
11	Scientific knowledge gain (‘Erkenntnisgewinn’)	155	–	29	–	119	–
12	Relevance to more sustainability	154	0.295**	32	–	116	0.359**
13	Development of implementable solutions for practice	157	0.401**	32	–	118	0.478**
14	Science-practice cooperation on an equal basis	158	0.284**	33	0.422*	119	0.263**
15	Sum of all criteria values	152	0.529**	33	0.582**	119	0.485**

Associations have been analyzed using Spearman´s rank-order correlation. Non-significant associations (indicated by ‘–’) are, for reasons of clarity, not depicted

*p < 0.05, **p < 0.01 (all two-tailed)

As we wanted to determine the extent to which the selected criteria can explain the overall perceived success of the recent TDR project, we, additionally, calculated a correlation for the sum of all criteria values. The results revealed a significant correlation (*r* = 0.529), indicating a relatively high, positive interrelation.

To determine the interplay between these criteria, we, additionally, undertook a categorical principal components analysis, which resulted in the extraction of three factors with an eigenvalue > 1. The rotated factor matrix (see Table 8) shows the loadings of each success criteria for each of the three factors. The highest correlation values were assigned to the corresponding factor. As factor 1 sums up criteria related to the quality of results and relevance to practice, we characterize factor 1 as criteria of ‘output performance’. Factor 2 then contains three criteria describing the quality of cooperation; thus, we interpret factor 2 as ‘process performance’. As three variables that are typical scientific reputation indicators have high loadings on factor 3, we interpret factor 3 as ‘career opportunities’.

**Table 8:** Rotated factor matrix showing factor loadings for each answer item

Factor matrix	Factor 1	Factor 2	Factor 3
Cronbach’s alpha	0.818099	0.731795	0.549536
	Output performance	Process performance	Career opportunity
Relevance to more sustainability	**0.943**	− 0.144	0.063
Development of implementable solutions for practice	**0.942**	− 0.148	0.063
Scientific knowledge gain (‘Erkenntnisgewinn’)	**0.837**	0.344	− 0.057
Transferability of results	**0.774**	0.293	− 0.110
Synthesis of results	**0.513**	0.294	0.132
Implementation of results into practice	**0.476**	0.463	0.141
Development of scientific methods and theories	**0.456**	0.436	0.341
Mutual learning	0.090	**0.863**	0.054
Representatives from all important stakeholder groups are involved	0.108	**0.852**	0.018
Science-practice cooperation on an equal basis	0.058	**0.635**	− 0.108
Scientific publication output	0.171	0.061	0.**825**
Provision of doctoral theses	0.172	− 0.078	0.**722**
Popularity of the project in the corresponding expert community	− 0.076	0.310	0.**640**
Acquisition of a follow-up project	0.112	0.156	− 0.307

Bold numbers show highest correlation values assigned to corresponding factor

Regarding each factor, the criteria with the highest loading act as a kind of proxy. It can thus be concluded that factor 1 (*r* = 0.295) and factor 2 (*r* = 0.299) have a similarly high association with overall perceived success perception, while factor 3 does not seem to have any relevant relation.

## Discussion

### What defines a successful transdisciplinary research project for participating scientists and practitioners?

According to our results, TDR is primarily considered successful when practical solutions for societal problems have been developed. This result stands regardless of the interviewees and survey respondents’ professional background, hierarchical standing in the project, and experience. Noticeably, all criteria with a high relevance to practice were rated especially important, while typically scientific success criteria were rated less important. Yet, the ideal-type concept of TDR advocated in literature contains the twofold objective emphasizing outcomes to practice and to science (see Lang et al. 2012; Bergmann et al. 2005; Jahn 2008; Belcher et al. 2016). In this regard, our finding indicates a significant imbalance within the science-practice outcome equilibrium with an orientation leaning toward the practice side of TDR. Even scientists, who were supposed to emphasize scientific success criteria based on their professional background, rated criteria of practical relevance higher. It could be argued that the framing of the interviews and questionnaire might have biased the responses, because societal relevance is a key distinguishing feature of TDR compared to (inter-)disciplinary research. However, the imbalance argument is supported by similar results from a previous study, in which we studied implementation obstacles of TDR in research practice. We found that a lack of conceptual clarity and a lack of knowledge about TDR among interview partners had been main explanatory factors that influence the diffusion of TDR. (Zscheischler et al. 2017; see also; Brandt et al. 2013; Jahn et al. 2012). Thus, it can be assumed that importance ratings of success reflect respondent`s understanding of TDR.

The practice orientation among scientists is remarkable: we expected success ratings to strongly reflect the success criteria sets of individual respondents and their respective professional and organizational background (cf. Serra and Kunc 2015; Cooke-Davies 2002). One explanation might be based on idealistic motivations, as the project’s contribution to a more sustainable use of natural resources was rated as highly important. Scientists thus seem to be highly committed to the transcending nature of TDR, which goes beyond the ‘ivory tower’. Such an interpretation would support the ‘dedifferentiation theory’ (Gibbons et al. 1994/2009; Nowotny et al. 2001). Furthermore, it illustrates the new role of ‘science as a change agent’ (Scholz 2017). Another explanation might be that scientists do not consider TDR to be ‘real’ scientific practice. This assumption is supported by the low importance ratings of the items ‘scientific publication output’ and ‘provision of doctoral theses’. From scientists’ perspective, specific scientific added value is not recognized. Either way, we argue that the potential for scientific knowledge gain from TDR remains unconsidered. Still, TDR-specific benefits for science seem to be vague (see also Zierhofer and Burger 2007).

In addition, the results illustrate ‘success as a multidimensional construct’ (McLeod et al. 2012). Eleven of fourteen items gathered from the qualitative interviews were rated as relatively important, with values higher than ‘3’ and even ‘4’, indicating their importance. Thus, quantitative results support the outcome from the qualitative interviews. This also indicates that the selected success criteria represent an adequate set to describe a TDR project according to the understanding of experienced researchers and practitioners. Differences between independent sample groups (differentiated into groups of age, disciplinary background, professional experience, and age) showed little significant variance regarding group preferences. We argue that this indicates a basic shared ‘success profile’ among most participants. This ‘success profile’ highlights key items such as ‘mutual learning’, ‘science-practice cooperation on an equal basis’ or ‘synthesis of results’ corresponding with items often mentioned in the TDR literature (Hirsch Hadorn et al. 2006, Mobjork 2010; Jahn 2008; Scholz and Steiner 2015; Zscheischler and Rogga 2015).

While the reluctant responses from the interviews with coordinating researchers might indicate that project performance is a rather less reflected issue, the responses from the online survey, additionally, suggest that a wide range of criteria are simultaneously important for the success of a TDR project (de Wit 1988; Bornmann and Marx 2012). Thus, the hesitant responses might reflect the complex interplay of success criteria. In contrast, hesitant and sparse answers may reflect not only conceptual deficiencies related to the idea of transdisciplinarity (Fuest and Lange 2015; Zscheischler et al. 2017) but also a lack of self-reflection processes about the quality and impact of the project.

### Perceived success and differences between the TDR ‘success profile’ and the recent TDR project

At the first glance, overall perceived success, with a mean value of 3.6 on a 5-point scale, can be regarded as somewhat moderate; it also indicates that potential for improvement remains. Deeper analysis shows that practitioners were less satisfied. Beyond that and contrary to our expectations, we found no differences between further independent sampling groups. We had hypothesized that individual interests, preferences, and targets vary depending on project experience, age, position (in project hierarchy), and disciplinary background and, thus, influence success ratings. This assumption has not been proven. Individual target systems seem to play a minor role. We, thus, conclude that there seems to be broad agreement on project objectives and high commitment regarding project goals.

Furthermore, we found a correlation between perceived performance during actual projects and the assessment of the general importance of these success criteria. On average, more important factors also score higher on the success scale (see Fig. 2). The data, however, do not reveal the direction of the correlation. Confirmation bias might play a role here, in the sense that ‘important is what performs well, respective to what I know as TDR from project experience’. Noticeably, no correlation could be found for three of the most highly rated criteria: ‘mutual learning’, ‘development of implementable solutions’, and ‘implementation of results into practice’. This may indicate that these criteria are widely uncontested and consolidated in the science-practice community.

In addition, there were several positive associations between the overall success assessment and criteria rating for the recent TDR project (see Table 7), which indicates that several criteria are simultaneously important and have to be considered for TDR projects to be conducted successfully. We assume that a deficit in fulfilling one criterion cannot be compensated by overperforming in another (see also Bornmann and Marx 2012) and argue that this assumption is supported by the broad correlation pattern with significant but moderate correlations. However, based on the association between the sum of all criteria values and the perceptions of overall success, we conclude that TDR-specific criteria can explain the perceived overall success of TDR projects only partially. We assume that there are additional important TDR-specific criteria, such as ‘collaboratively framing the research problem’ (e.g., Lang et al. 2012), that we did not consider in this study. Non-TDR-specific criteria may also play a critical role. Studies on success perception from the field of management research could demonstrate that success criteria vary widely among stakeholder groups in projects going beyond those projects’ committed criteria (Davis 2014). Thus, the identification of a complete set of success criteria is unlikely to be achievable (de Wit 1988).

In addition, quality criteria related to team cooperation can be reasonably assumed to be crucial for the perception of success in TDR projects. Cognitive, emotional, and interactional dimensions (Boix Mansilla et al. 2016) such as ‘personal chemistry’ have shown to be influential (Tress et al. 2007). Only a few of the criteria investigated in our study (‘science-practice cooperation on an equal basis’ and ‘mutual learning’) point to the social level of TDR projects. An additional criterion which we suggest might be ‘project performance’, meaning that perceived project success might be shaped by the bare completion of project steps (‘milestones’), as planned in the project proposal. This success criterion must not be underestimated, especially for staff members in coordinating positions.

As already mentioned, practitioners tended to have moderate success ratings. One explanation can be found in the poor project performance related to the criteria previously rated as most important: ‘development of implementable solutions for practice’ and ‘implementation of results into practice’. In contrast, it is notable that these items have no significant relation to overall perceived success. In contrast, the two criteria ‘popularity of the project’ and ‘acquisition of a follow-up project’ are correlated quite strongly with overall perceived success. Yet, these criteria were rated to have lower importance in the ‘TDR success profile’ (see Table 4). A possible explanation may relate to a target shift that might have taken place during the course of the project (see also de Wit 1988; Meyer 1994). Thus, when practitioners realized that their main targets were not achievable, they shifted to other benefits on a rather personal and organizational level: the ‘popularity of the project’ increases one’s own reputation, while the ‘acquisition of a follow-up project’ facilitates organizational development and financing acquisition. In addition, there were also high associations with the criteria ‘mutual learning’ and ‘cooperation on an equal basis’, which indicates that the quality of the cooperation also offers specific benefits to practitioners and is especially important to their perceived project success.

Using categorical principal components analysis, we determined the extent to which the criteria are interrelated which each other. Our results showed that there is an interplay within different criteria, resulting in the extraction of three factors: ‘output performance’, ‘process quality’, and ‘career opportunities’. While we did not find associations for career opportunities, the two factors ‘output performance’ and ‘process quality’ appear to have the same level of importance for the overall perceived success of TDR projects. This finding confirms the outstanding role of process quality for TDR projects, as argued by many authors (see Lang et al. 2012). In addition, these factors reflect typical concepts for projects success, as described in project management literature: management success and product success (see Baccarini 1999).

### Implications

In our study, we tested a set of criteria for the relevance of success perceptions that widely overlaps with other sets of quality criteria (e.g., Bergmann et al. 2005; Lang et al. 2012; Wolf et al. 2013; Belcher et al. 2016) but were reduced and selected by the need to be understandable and assessable by a broader community of researchers and practitioners. Results showed that a multitude of criteria are considered but are weighted differently by scientists and practitioners. We argue that these individual perspectives are of substantial interest and should be considered.The different weighting of the criteria shows that relevance for practice currently receives greater emphasis than scientific relevance. We consider this imbalance quite critically, as TDR should cover more than ‘consultancy’ or ‘societal practice’ (see also Rohe 2015), and we thus argue that the scientific knowledge gain from TDR projects holds an important potential for the ability to answer urgent real-world problems that demand an orientation for action and a high degree of normativity and value setting. Research may provide an orientation for action and involve actors from practice, but it still must be in accordance with the foundations of scientific excellence to substantiate practice solutions with evidence. However, at the moment, this issue does not seem to be adequately considered in TDR projects. We argue that the debate about quality criteria for TDR must pay more attention to the specific quality of scientific practice and outputs from TDR projects in the future. At the moment, we cannot find concrete quality criteria from the literature that reflect this issue in a more detailed manner.

A measurable quality increase in scientific results from TDR projects also supports the legitimacy of TDR, as the approach itself is ‘not uncontested’ (see Lang et al. 2012).

Regarding the reluctant response behavior of coordinators—and based on knowledge from a number of proposals—we noticed that self-reflective procedures were rarely applied in TDR projects. Yet, we think that a high potential for learning, quality management, and success of TDR can be found in subsequently conducted self-evaluation (see also Bergmann et al. 2005).

At the moment, the evaluation of science is especially shaped by expert perspectives, bibliographic output analyses, and quantitative indicators of cooperation (e.g., number of workshops and participants), which are proxies and hardly meaningful for measuring the quality of a project and its science. Although individual ratings are very subjective and composed of myriad other influencing factors, we argue that a success assessment by participating researchers and practitioners can give insights into the quality of TDR.

### Methodological approach

For this study, we combined qualitative interviews with an online survey. The two data sources provided a good opportunity to gather complementary information. It proved a useful approach to achieving the goals of this study.

We acknowledge that the qualitative interviews only covered the scientific perspective. It was especially difficult to access practitioners, because their availability for the projects was limited. This is also illustrated by the relatively low number of practitioners (*n* = 45) who participated in the survey. Thus, we cannot exclude a strong participation bias. However, the previous studies completely disregarded practitioners’ perspectives on TDR. It would be of special interest to gather such information.

Another aspect relates to the funding context. All the respondents were involved in projects funded by the same program. Thus, our results also reflect a specific notion of TDR that was reasonably influenced by the call for applications. The funding program focused on practical relevance. Thus, our results only cover the specific research field of land-use science in which applied scientists and engineers are in the majority.

Furthermore, uncertainty about the role of the SCP persisted amongst the project researchers. There has been an ongoing fear of being controlled and of having ideas stolen; this fear could only be partially controlled throughout the duration of the project.

As a result, we decided not to ask for the projects’ names, thus avoiding further concern on the part of the researchers about being evaluated by the SCP. Therefore, our results do not relate to single projects, which surely would have provided promising insights.

## Conclusion

In this study, we aimed to obtain information about the perceived success of TDR from scientists and practitioners with experience with TDR projects in the domain of land-use science. We built on the observation that most attempts to develop adequate evaluation approaches for TDR are expert driven and related to an idealized TDR concept. In contrast, knowledge about individual success perception amongst a broader community of applying researchers and practitioners remains rare. We argue that their perspectives are of special interest, as TDR can be regarded as a new form of scientific practice. Further adoption and dissemination will also rely on the attitudes and inclinations toward TDR.

By conducting qualitative interviews with coordinating scientists, complemented by a literature review, we gathered 14 criteria reflecting TDR success dimensions that are largely understandable and assessable by scientists and practitioners. In an online survey, we received 178 completed questionnaires from scientists and practitioners who rated the criteria set first for general importance regarding TDR and second for succeeding in their recent TDR project.

Our study showed that the assessment of TDR project success is a complex issue and that, to date, it rarely follows formative self-evaluation procedures. However, the survey identified a basic shared ‘success profile’ among both scientists and practitioners, reflecting a consolidating concept of TDR in the application field of land-use science.

Nevertheless, this ‘success profile’ shows a significant imbalance within the science-practice outcome equilibrium, as advocated in the literature, with an orientation leaning toward the practice side of the TDR ideal. The specific scientific benefits of TDR do not seem to be recognized, thus indicating that TDR’s considerable potential remains neglected.

The overall perception of the success of TDR projects can be described as relatively moderate, indicating several deficits in the application and management of TDR. Professionalization of TDR management is, nevertheless, pending. We also found that a multitude of criteria are simultaneously important to the overall success perceptions of TDR projects. Furthermore, additional criteria not related to TDR are assumed to play an important role, as overall success perceptions cannot be explained completely by the tested criteria.

Notably, although perceptions of overall project success are strongly associated with process quality and output performance, personal targets, such as career opportunities, seem to have little influence. We conclude that there is especially high commitment to project targets.

## Electronic supplementary material

Below is the link to the electronic supplementary material.

Supplementary material 1 (DOCX 45 KB)
