# Supplementary material for: The success of transdisciplinary research for sustainable land use: individual perceptions and assessments
Source: Sustain Sci. 2018 Mar 28;13(4):1061–74. doi: 10.1007/s11625-018-0556-3 (PMC6086303; doi:10.1007/s11625-018-0556-3)
Supplement: Supplementary file 1 — Supplementary material 1 (DOCX 45 KB) [file 11625_2018_556_MOESM1_ESM.docx]

**Supplementary Information. Success of Transdisciplinary Research for Sustainable Land Use - individual Perceptions and Assessments**

1. **The following quotes are referenced in section 3:**

| QNo. | Quote |
| --- | --- |
| *Q1* | *“Is there an added value? That is a difficult question. I think, generally, Yes, there is. [hesitates] I am pondering because the answer to that question is not very satisfying.”* |
| *Q2* | *“It is vague; it is difficult too....”* |
| *Q3* | *“I cannot exactly quantify the share from the project: 80 %, 20 %, 30 %, 70 %,… well, you see, I can’t tell. As an example, short rotation coppices have been implemented, we provided economical and feasibility studies and so on, and I would really state, that the project holds a big share. Still, this formal implementation happened after the end of the project...two, three months after, but the largest share of the way was paved by the project.”* |
| *Q4* | *“I can hardly assess whether the results are implementable, can be implemented or are even helpful; I don’t know.”* |
| *Q5* | *“I think it is a very subjective question, too. Partly, you have a couple of objective criteria on hand, but one person recognizes success differently than the other within the same project.”* |
| *Q6* | *“Everyone who speaks about that project sees how great it is and what results we achieved, and considering the area of conflict and the difficulty of inter- and transdisciplinary challenges, we have accomplished a lot, didn’t we?”* |
| *Q7* | *“Of course, you have different senses depending on the project phase you’re in. You (either) think the whole project is miserable, or you experience moments of joy, oah, that’s super, that is a super result.”* |

1. **Sample Characteristics:**


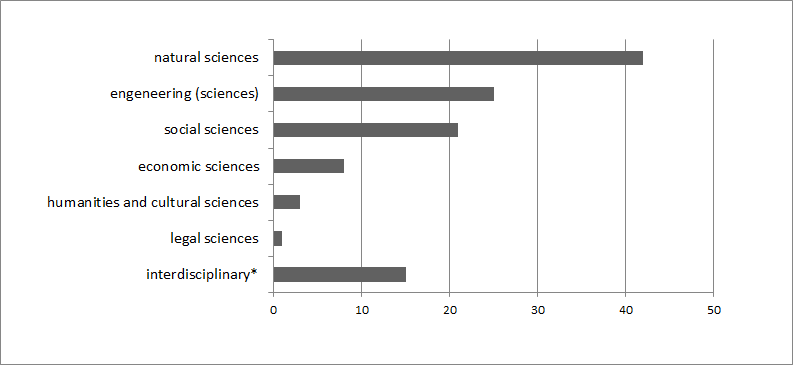


Figure 1.Disciplinary background of participating scientists (number of assignments). * scientists who belong to natural sciences or engineering and one of the other disciplines were classified as ‘interdisciplinary’


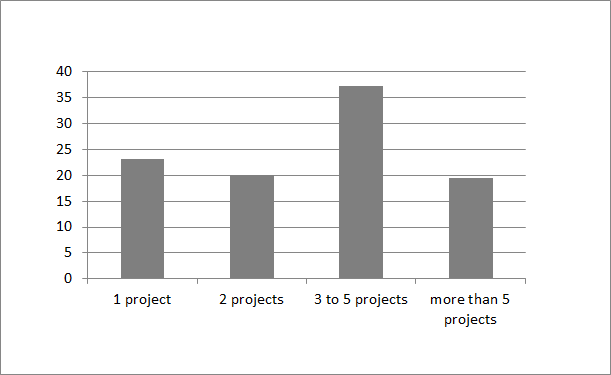


Figure 2. Project experience of respondents. Question: In how many transdisciplinary research projects have you already participated?


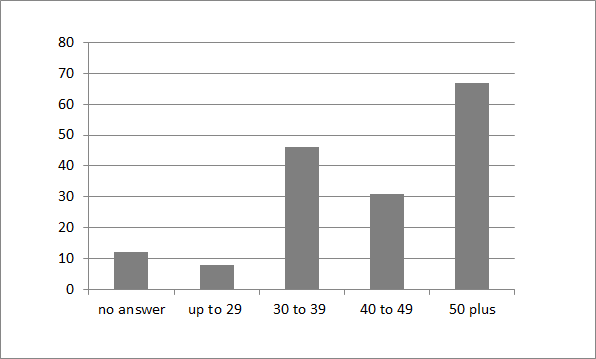


Figure 3. Age of respondents. Question: Which age group are you in?
